# Supplementary material for: MMAB promotes negative feedback control of cholesterol homeostasis
Source: Nat Commun. 2021 Nov 8;12:6448. doi: 10.1038/s41467-021-26787-7 (PMC8575900; doi:10.1038/s41467-021-26787-7)
Supplement: Supplementary file 1 — Supplementary Information [file 41467_2021_26787_MOESM1_ESM.pdf]

SUPPLEMENTARY INFORMATION FILE

MMAB promotes negative feedback control of cholesterol homeostasis

Leigh Goedeke[1,2], Alberto Canfrán-Duque[1,3], Noemi Rotllan[1,3], Balkrishna Chaube[1,3], Bonne M. Thompson[4], Richard G. Lee[5], Gary W. Cline[2], Jeffrey G. McDonald[4], Gerald I. Shulman[2,6], Miguel A. Lasunción[7], Yajaira Suárez[1,3], and Carlos Fernández-Hernando[1,3,#]

[1]Vascular Biology and Therapeutics Program, Yale School of Medicine
[2]Department of Internal Medicine, Yale School of Medicine,
[3]Integrative Cell Signaling and Neurobiology of Metabolism Program, Department of Comparative Medicine and Pathology, Yale School of Medicine, New Haven CT 06520, USA
[4]Center for Human Nutrition. University of Texas Southwestern Medical Center, Dallas TX 75390, USA.
[5]Cardiovascular Group, Antisense Drug Discovery, Ionis Pharmaceuticals, Carlsbad, CA, 92010, USA.
[6]Department of Cellular & Molecular Physiology, Yale School of Medicine
[7]Servicio de Bioquímica-Investigación, Hospital Universitario Ramón y Cajal, Instituto Ramón y Cajal de Investigación Sanitaria (IRyCIS) and CIBER de Fisiopatología de la Obesidad y Nutrición (CIBERobn), Madrid Spain.

[#]**Correspondence:** carlos.fernandez@yale.edu

This PDF file contains:

Supplementary Figure Legends
Supplementary Figures 1–9
Supplementary Table 1

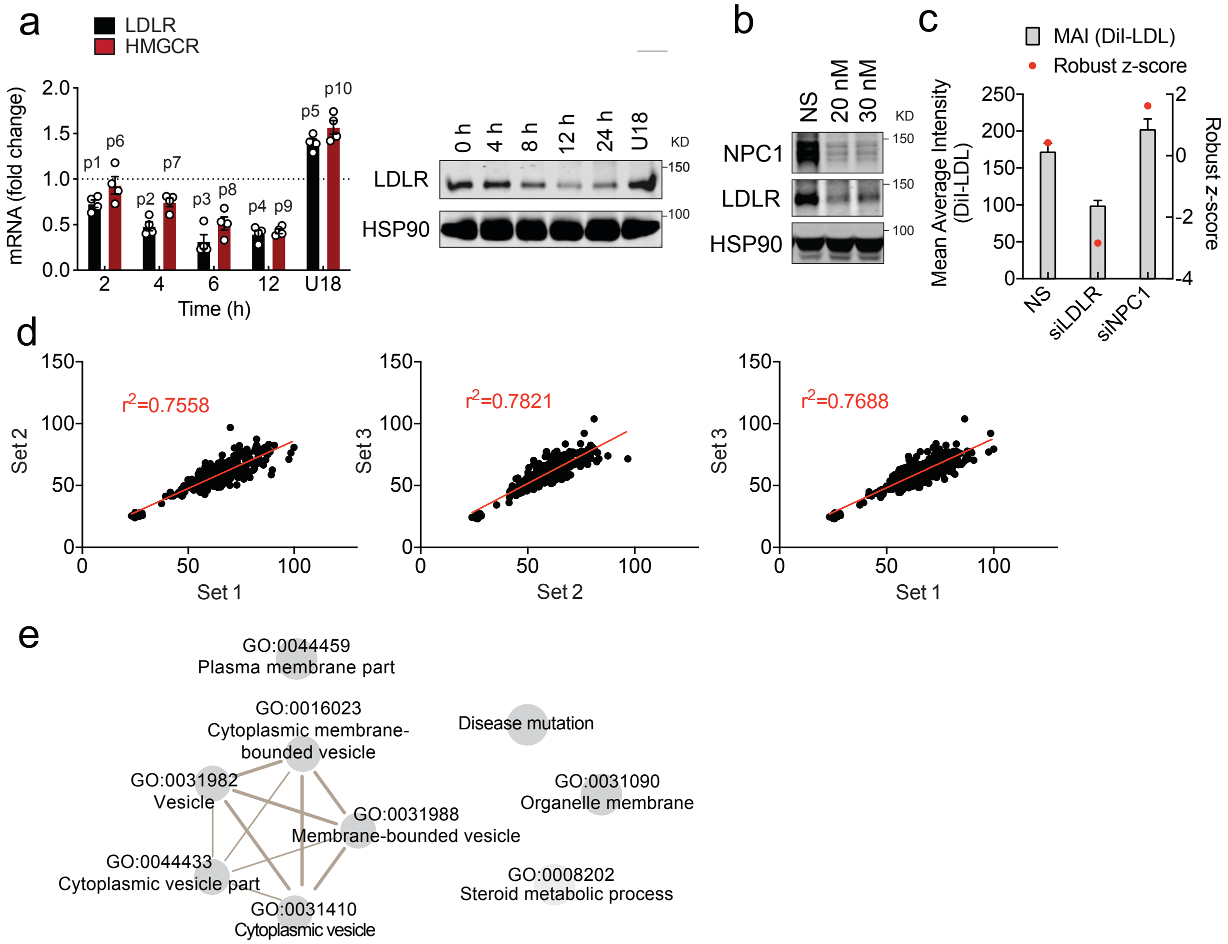

Supplementary Fig. 1: Screening controls using in primary RNAi screen. (**a**) qRT-PCR (*left panel*) and representative Western blot (*right panel*) analysis of HMGCR and LDLR in Huh7 cells treated with 30 µg/ml native LDL (nLDL) or U18666A (U18) for the indicated times. HSP90 was used as a loading control. Data are the mean ± s.e.m. of four independent experiments. Comparisons by two-sided unpaired Student's *t*-test compared to vehicle-treated cells (dashed line). p1= 0.02, p2= 0.001, p3= 0.0006, p4= 0.0003, p5= 0.003, p6= 0.46, p7= 0.01, p8= 0.002, p9= 0.0001, p10= 0.001. (**b**) Representative Western blot analysis of NPC1 and LDLR in Huh7 cells transfected with 20 and 30 nM of siRNA NPC1 (*top panel*), siRNA LDLR (*middle panel*) or non-silencing siRNA (NS). HSP90 (*bottom panel*) was used as a loading control. (**c**) Comparison of DiI-LDL mean average intensity (grey bars, MAI) and robust z-score (red dots) for Huh7 cells transfected with a non-silencing siRNA (NS), negative control siRNA (siRNA to LDLR, siLDLR) or positive control siRNA (siRNA to NPC1, siNPC1). Data are the mean ± s.e.m. (*n* = 128 technical replicates). (**d**) Representative linear regression analysis of DiI-LDL mean average intensity (MAI) for plate sets 1 and 2 (left), plate sets 2 and 3 (middle) and plate sets 1 and 3 (right). The goodness-of-fit (r^2) and regression line (indicative of overall reproducibility of the screen) are indicated in red on each graph. Each black dot corresponds to one well (*n* = 384). (**e**) Interaction among top GO terms identified from primary RNAi screen using the STRING interaction database and Cytoscape plugin. Source data are provided as a Source Data file.

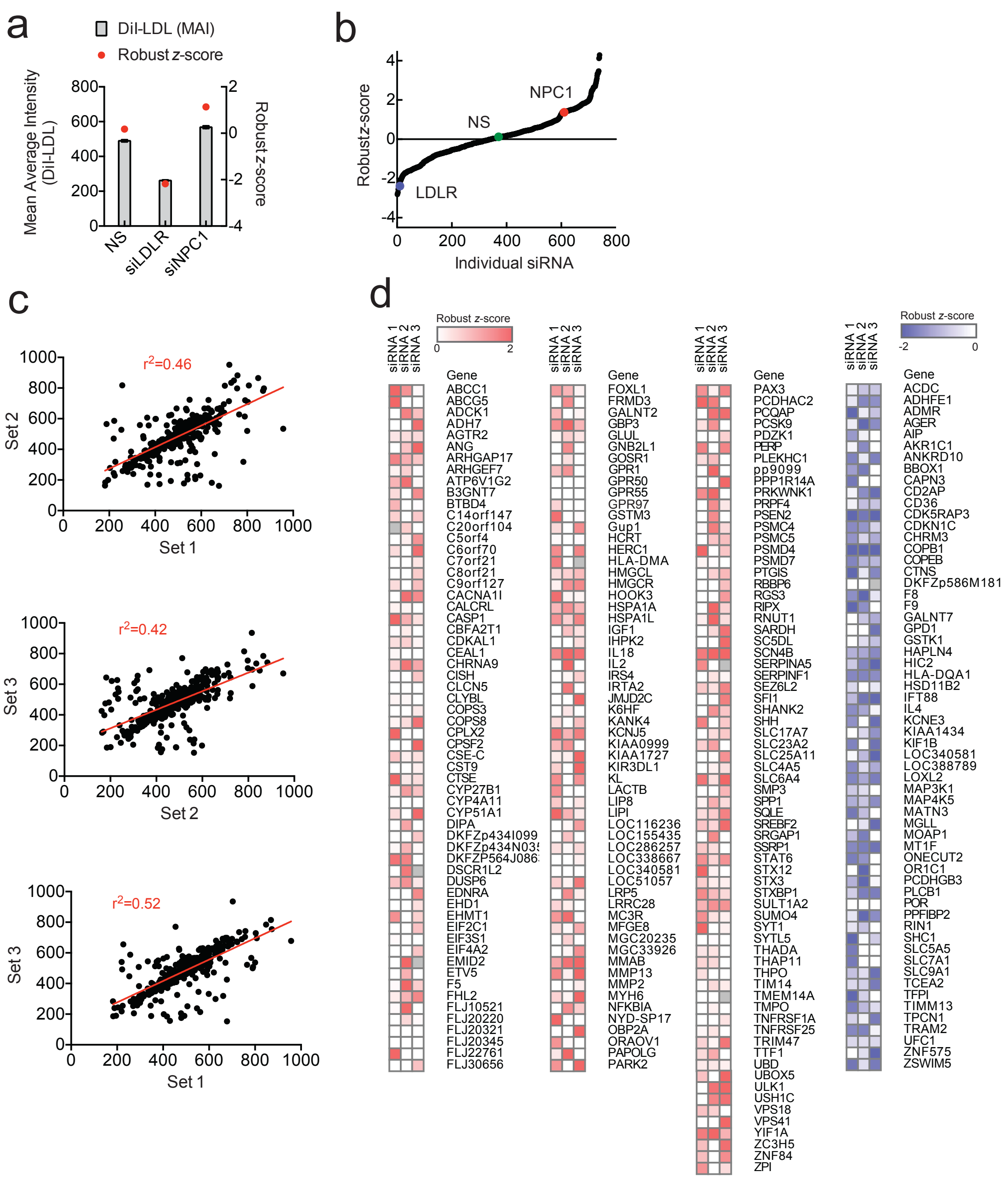

Supplementary Fig. 2: Primary RNAi screen validation. (**a**) Comparison of DiI-LDL mean average intensity (grey bars, MAI) and robust z-score (red dots) for Huh7 cells transfected with a control siRNA (non-silencing, NS), negative control siRNA (siRNA to LDLR, siLDLR) or positive control siRNA (siRNA to NPC1, siNPC1) in the deconvolution screen. Data are the mean ± s.e.m. (n = 90 technical replicates). (**b**) Distribution of average robust z-scores for individual siRNAs in the deconvolution screen (n = 784). Controls are NS siRNA (green dot), siLDLR (blue dot) and siNPC1 (red dot). All other siRNAs are shown in black. (**c**) Linear regression analysis of DiI-LDL mean average intensity for plate sets 1 and 2 (*top*), plate sets 2 and 3 (*middle*) and plate sets 1 and 3 (*bottom*). The goodness-of-fit (r^2) and regression line (indicative of overall reproducibility of the screen) are indicated in red on each graph. Each black dot corresponds to one well (n = 384). (**d**) Heatmap of genes validated in the deconvolution screen. Three siRNAs per gene used in the primary RNAi screen were assayed individually in 384-well plates using the same screening strategy outlined in **Fig. 1a**. Source data are provided as a Source Data file.

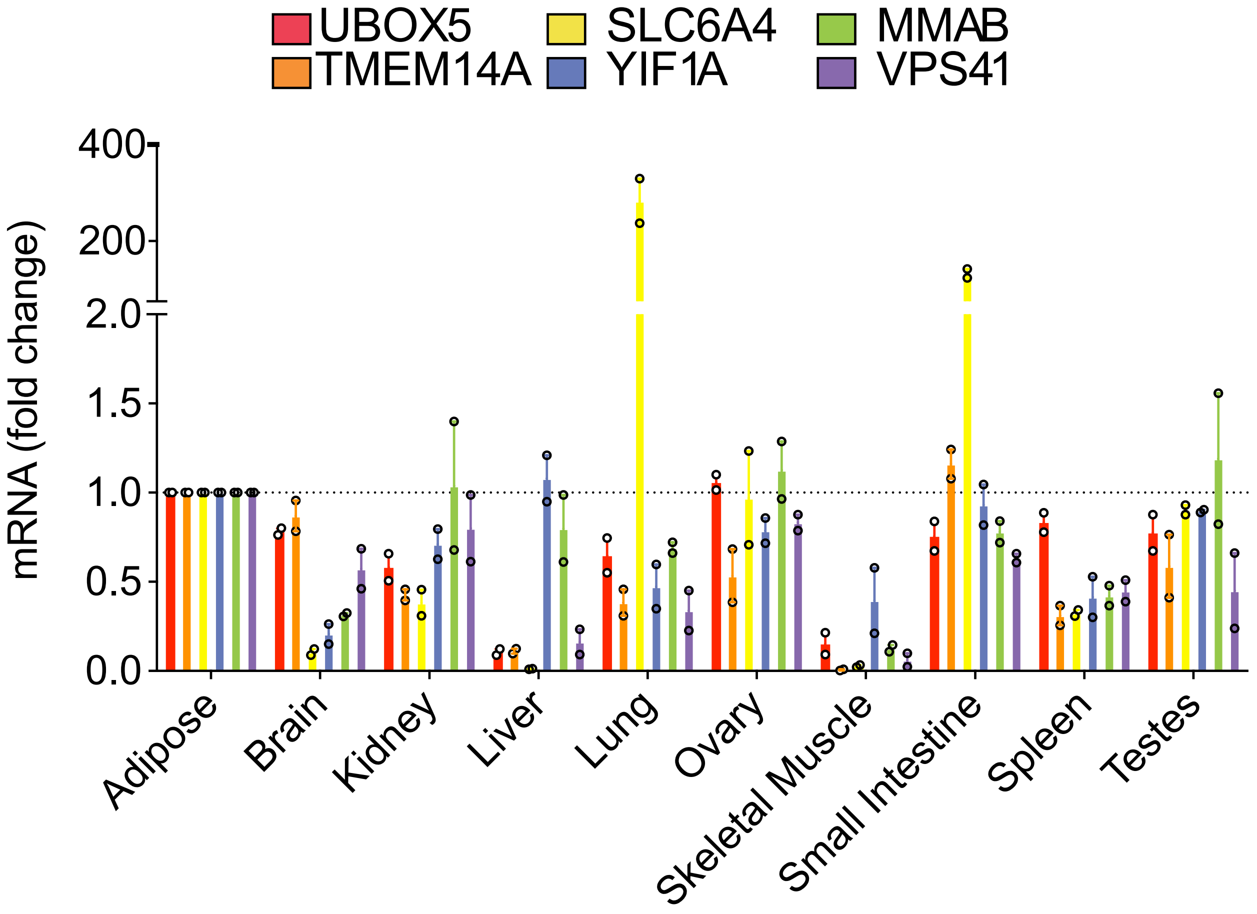

Supplementary Fig. 3: Tissue expression of top candidate genes from RNAi screen. qRT-PCR analysis of *UBOX5*, *SLC6A4*, *MMAB*, *TMEM14A*, *YIF1A*, and *VPS41* in selected human tissues (Life Technologies). Data are expressed as fold-change relative to adipose tissue and the mean ± s.e.m. of two independent experiments. Source data are provided as a Source Data file.

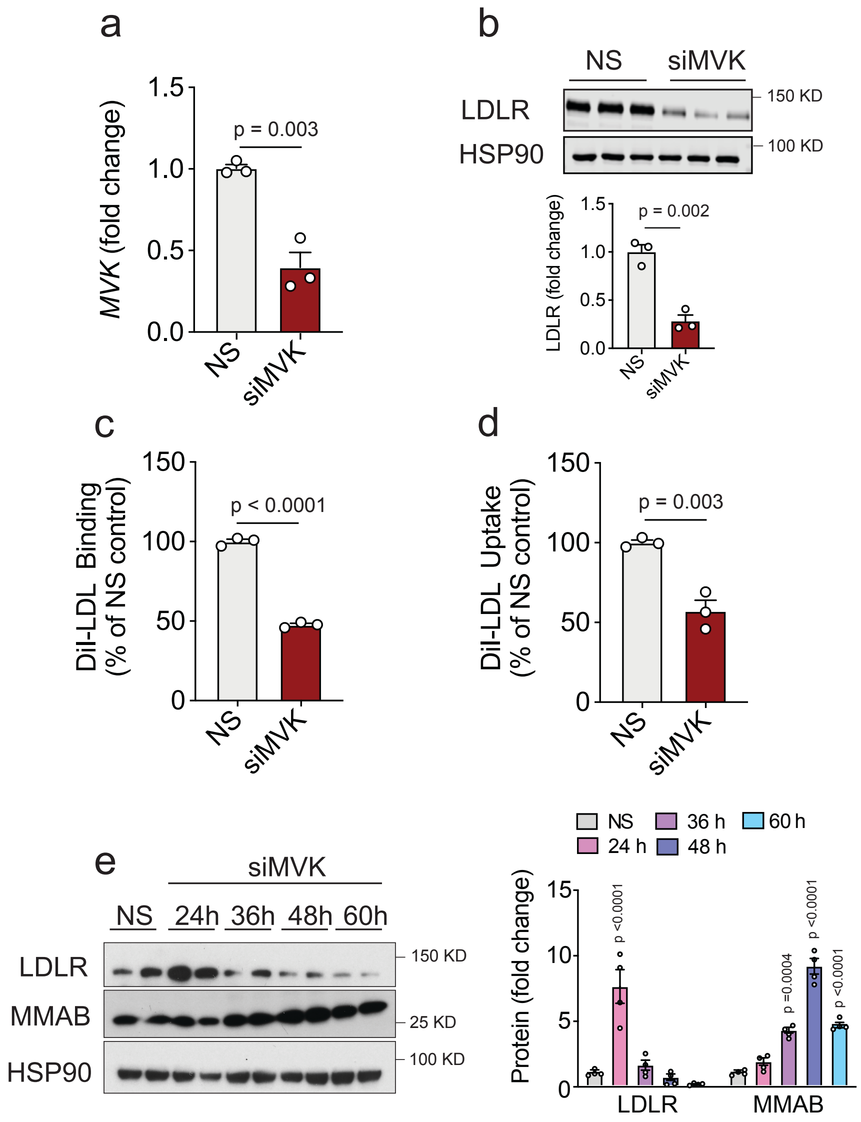

Supplementary Fig. 4: Knockdown of MVK decreases LDLR expression and activity. (a) qRT-PCR analysis of *MVK* expression in Huh7 cells transfected with a siRNA against MVK (siMVK) or non-silencing control siRNA (NS) for 60 h. Data are the mean ± s.e.m. of three independent experiments in duplicate. Statistical comparisons between groups by two-tailed unpaired Student's *t*-test. (**b**) Representative Western blot analysis of LDLR expression in Huh7 cells treated as in (**a**). HSP90 was used as a loading control. Quantification of blot is shown below. Data are the mean ± s.e.m. of three independent experiments in triplicate. Statistical comparisons between groups by two-tailed unpaired Student's *t*-test. (**c**–**d**) Flow cytometry analysis of DiI-LDL specific binding (**c**) and uptake (**d**) in Huh7 cells transfected with NS siRNA or siMVK for 60 h and incubated with 30 µg/ml DiI-LDL for 2 h at 37 °C (uptake) or 90 min at 4 °C (binding). Data are the mean ± s.e.m. of three independent experiments in triplicate. Statistical comparisons between groups by two-tailed unpaired Student's *t*-test. (**e**) Representative Western blot analysis of LDLR and MMAB expression in Huh7 cells transfected with a siRNA against MVK (siMVK) or non-silencing control siRNA (NS) for 24–60 h. HSP90 was used as a loading control. Quantification of blots is shown to the right. Data are the mean ± s.e.m. of four independent experiments in duplicate. Statistical comparisons between groups by two-way ANOVA with Bonferroni correction for multiple comparisons (**e**). Source data are provided as a Source Data file.

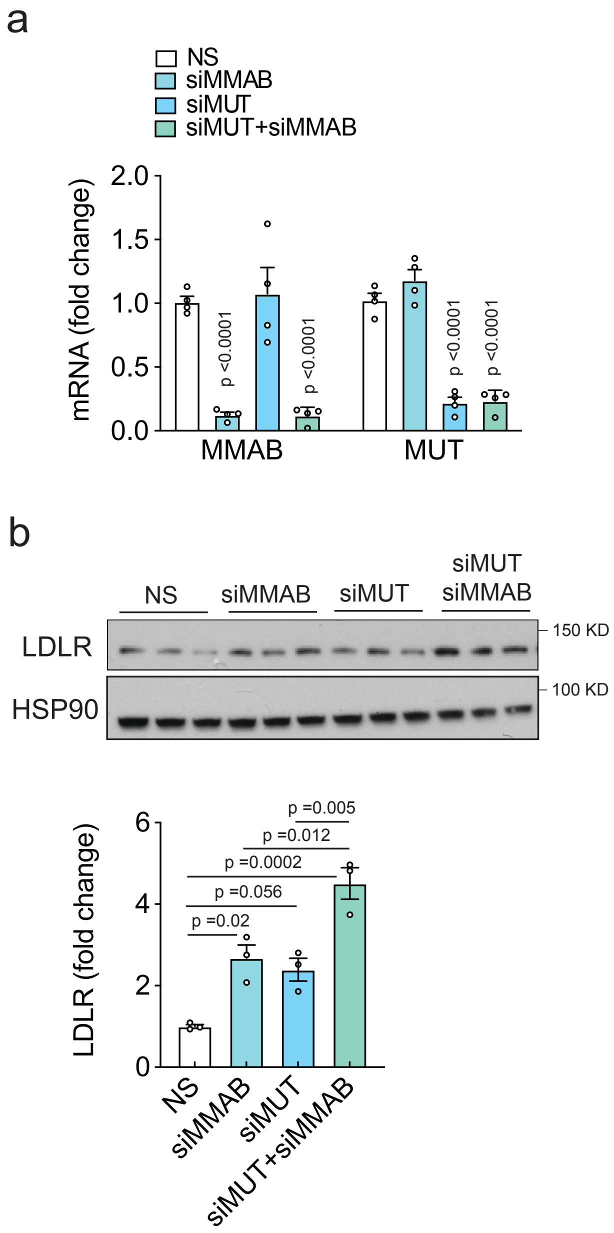

Supplementary Fig. 5: Knockdown of MMAB and MUT increase LDLR expression. (a) qRT-PCR analysis of *MMAB* and *MUT* expression in Huh7 cells transfected with a siRNA against MMAB (siMMAB), MUT (siMUT) or siRNA against MUT and MMAB (siMUT+MMAB). Data are the mean ± s.e.m. of four independent experiments in duplicate. Statistical comparisons between groups by two-way ANOVA with Bonferroni correction for multiple comparisons. (**b**) Representative Western blot analysis of LDLR expression in Huh7 cells treated as in (**a**). HSP90 was used as a loading control. Quantification of blots is shown below. Data are the mean ± s.e.m. of three independent experiments in triplicate. Statistical comparisons between groups by one-way ANOVA with Bonferroni correction for multiple comparisons. Source data are provided as a Source Data file.

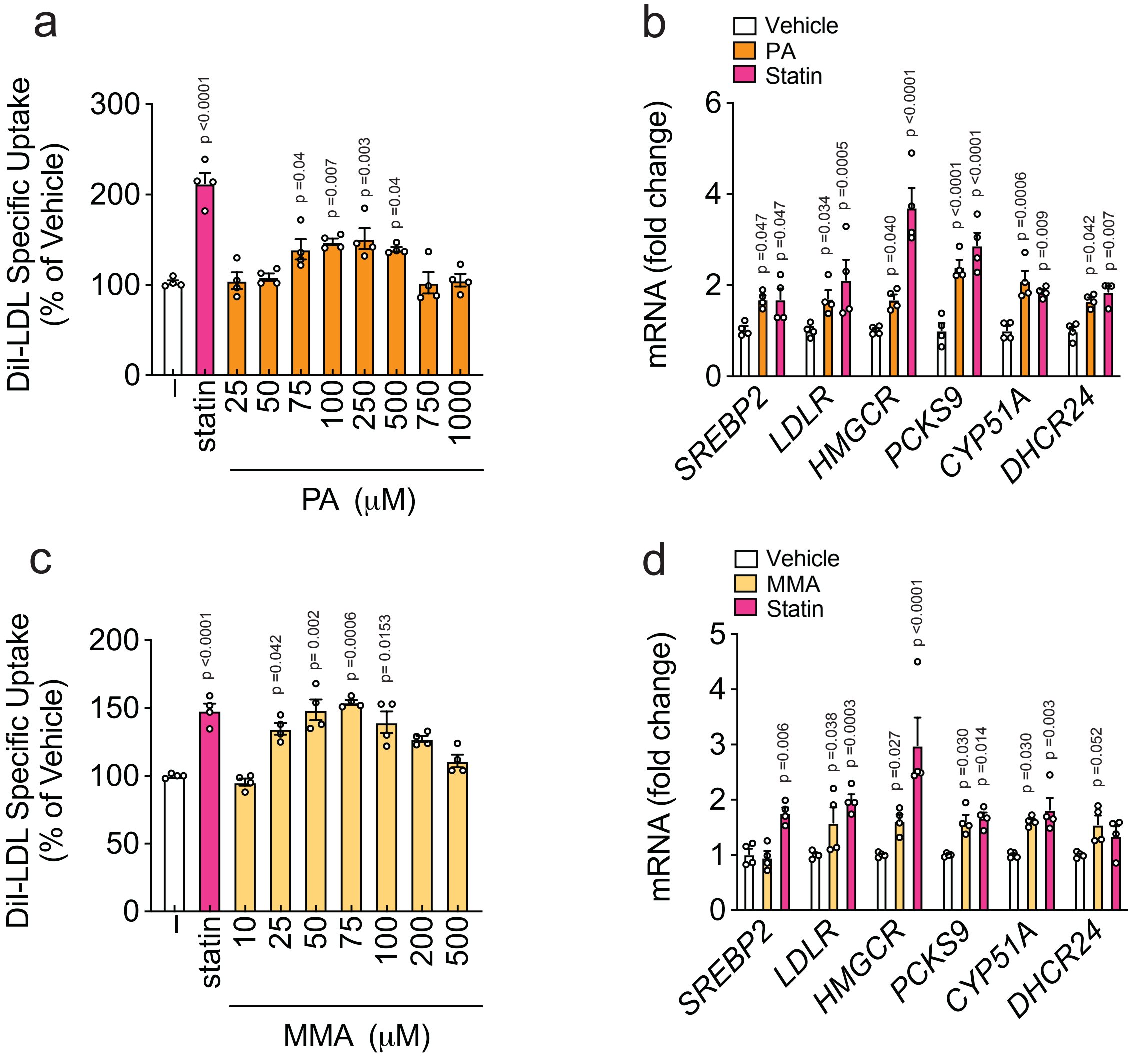

Supplementary Fig. 6: Propionic and methylmalonic acid treatment increases DiI-LDL specific uptake and SREBP2-mediated gene expression in Huh7 cells. (**a**) Flow cytometry analysis of DiI-LDL specific uptake in Huh7 cells treated with vehicle (–), 5 μM statin (statin), or the indicated doses of propionic acid (PA) for 24 h and incubated with 30 μg/ml DiI-LDL for 2 h at 37 °C. Data are the mean ± s.e.m. of three independent experiments in triplicate. Statistical comparisons between groups by one-way ANOVA with Bonferroni correction for multiple comparisons. (**b**) qRT-PCR analysis of SREBP2-responsive genes in Huh7 cells incubated in LPDS for 24 h and treated with vehicle (–), 100 μM propionic acid (PA) or 5 μM statin (ST) for an additional 24 h. Data are the mean ± s.e.m. of three independent experiments in duplicate. Statistical comparisons between groups by one-way ANOVA with Bonferroni correction for multiple comparisons. (**c**) Flow cytometry analysis of DiI-LDL specific uptake in Huh7 cells treated with vehicle (–), 5 μM statin (statin), or the indicated doses of methylmalonic acid (MMA) for 24 h and incubated with 30 μg/ml DiI-LDL for 2 h at 37 °C. (**d**) qRT-PCR analysis of SREBP2-responsive genes in Huh7 cells incubated in LPDS for 24 h and treated with vehicle (–), 50 μM methylmalonic acid (MMA) or 5 μM statin (ST) for an additional 24 h. Data are the mean ± s.e.m. of four independent experiments in duplicate. Statistical comparisons between groups by one-way ANOVA with Bonferroni correction for multiple comparisons. Source data are provided as a Source Data file.

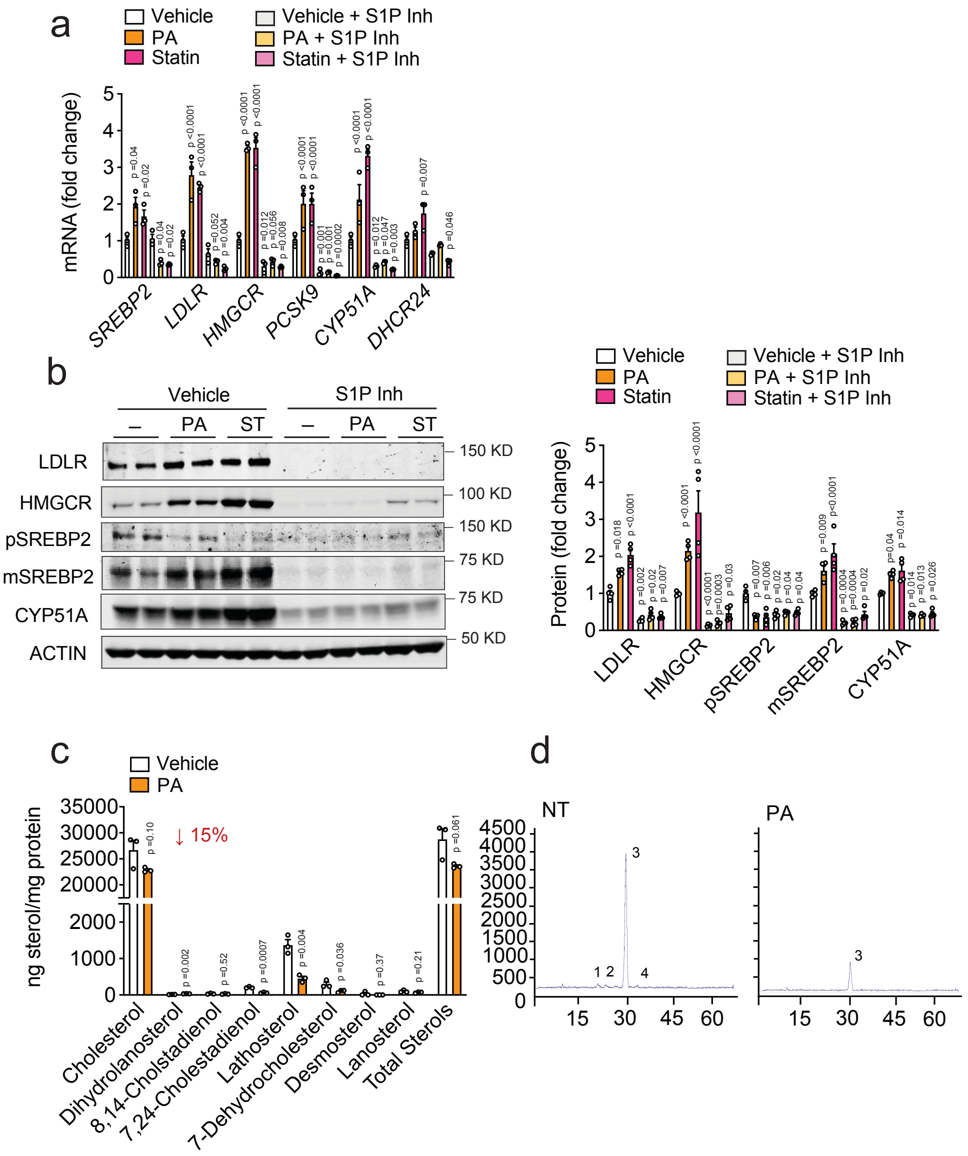

Supplementary Fig. 7: Propionic acid treatment decreases total sterol content. (**a–b**) qRT-PCR (**a**) and Western blot analysis (**b**) of SREBP2-responsive genes in Huh7 cells incubated in LPDS for 24 h and treated with vehicle (–), 100 µM propionic acid (PA) or 5 µM statin (ST) for an additional 24 h with or without an S1P inhibitor. Actin was used as a loading control. Quantification of blots shown to the right (**b**). Data are the mean ± s.e.m. of three independent experiments in duplicate (**a**) or four independent experiments in duplicate (**b**). Statistical comparisons between groups by one-way ANOVA with Bonferroni correction for multiple comparisons. (**c**) Sterol content of Huh7 cells pretreated in LPDS for 24 h and treated with vehicle (–) or 100 µM propionic acid (PA) for 24 h. Lipids were extracted and analyzed by GC-MS. "Total sterols" were calculated as the sum of all sterols that could be detected. Data are the mean ± s.e.m. of three independent experiments. Statistical comparisons between groups by two-tailed unpaired Student's *t*-test. (**d**) Effect of propionic acid on [1,2-^{14}C]-acetate incorporation into sterols as determined by HPLC. Cells were treated as in (**c**). 1, desmosterol; 2, 7-dehydrocholesterol; 3, cholesterol; 4, lanosterol. Data are representative of three independent experiments. Source data are provided as a Source Data file.

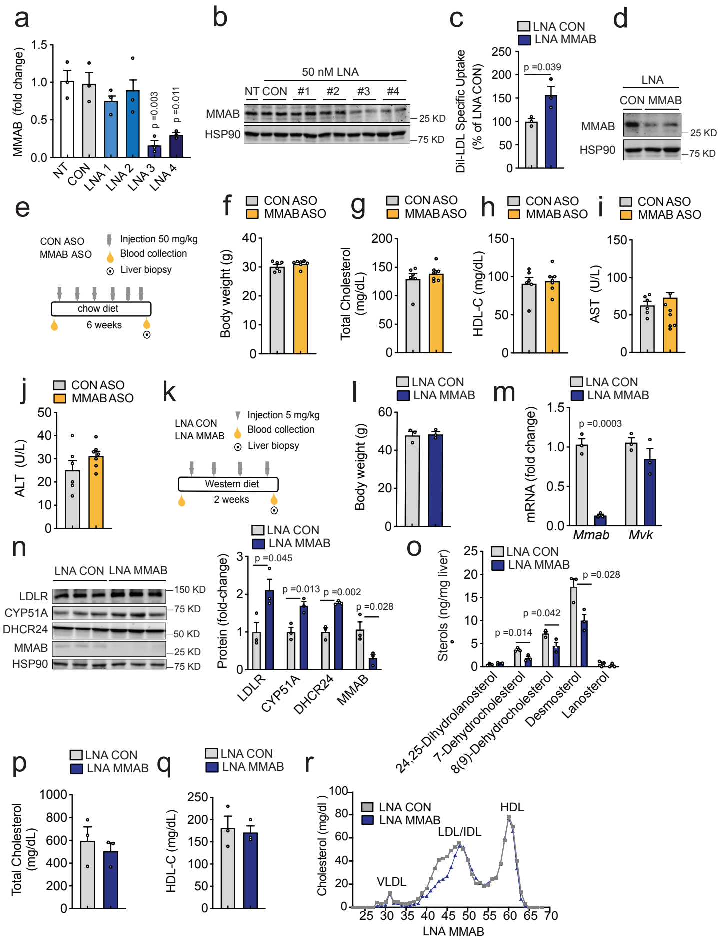

Supplementary Fig. 8: Knockdown of MMAB increases LDLR expression *in vivo*. (**a-b**) qRT-PCR (**a**) and representative Western blot analysis (**b**) of MMAB expression in mouse hepatic (Hepa) cells transfected with 50 nM of control LNA (LNA CON) or 50 nM of 4 different LNA GapMers against MMAB (LNA #1–4). NT, non-transfected. HSP90 was used as a loading control. Data are the mean ± s.e.m. of three independent experiments in duplicate. Statistical comparisons between groups by one-way ANOVA with Bonferroni correction for multiple comparisons. (**c**) Flow cytometry analysis of DiI-LDL uptake in Hepa cells transfected with 50 nM of a control LNA (LNA control) or an LNA GapMer against MMAB (LNA MMAB). Representative Western blot showing MMAB knockdown is shown in panel (**d**). CON, control LNA; MMAB, LNA MMAB. Data are the mean ± s.e.m. of three independent experiments. Statistical comparisons between groups by two-tailed unpaired Student's *t*-test. (**e**) Experimental outline of MMAB ASO treatment in mice. 8-week old male C57BL/6 mice were fed a chow diet and treated once weekly with 50 mg/kg control ASO (CON ASO, $n = 6$) or 50 mg/kg MMAB ASO ($n = 7$) for six weeks. (**f–j**) Body weight (**f**), total cholesterol (**g**), HDL-C (**h**), AST (**i**), and ALT(**J**) in mice treated as in (**e**). Data are the mean ± s.e.m. (**k**) Experimental outline of MMAB LNA treatment in mice. 8-week old male *Apobec1*$^{-/-}$*;Ldlr*$^{+/-}$mice were fed a Western diet for 2 months and then treated with 5 mg/kg LNA control or LNA MMAB for two weeks ($n = 3$ per group). (**l**) Body weight of mice treated as in (**k**). Data are the mean ± s.e.m. (**m–n**) qRT-PCR (**m**) and Western blot (**n**) analysis of MMAB and SREBP2-responsive genes in the livers of mice treated as in (**k**). HSP90 was used a loading control. Quantification of blot shown to the right. Data are the mean ± s.e.m. Statistical comparisons between groups by two-tailed unpaired Student's *t*-test. (**o**) Hepatic sterol content in the livers of mice treated as in (**k**). Lipids were extracted and analyzed by GC-MS. $n = 3$ per group. Data are the mean ± s.e.m. Statistical comparisons between groups by two-tailed unpaired Student's *t*-test. (**p–q**) Total cholesterol (**p**) and HDL-C (**q**) in mice treated as in (**k**). Data are the mean ± s.e.m. (**r**) Cholesterol content of FPLC-fractionated lipoproteins from pooled plasma (n =3 per group) of mice treated as indicated in (**k**). Source data are provided as a Source Data file.

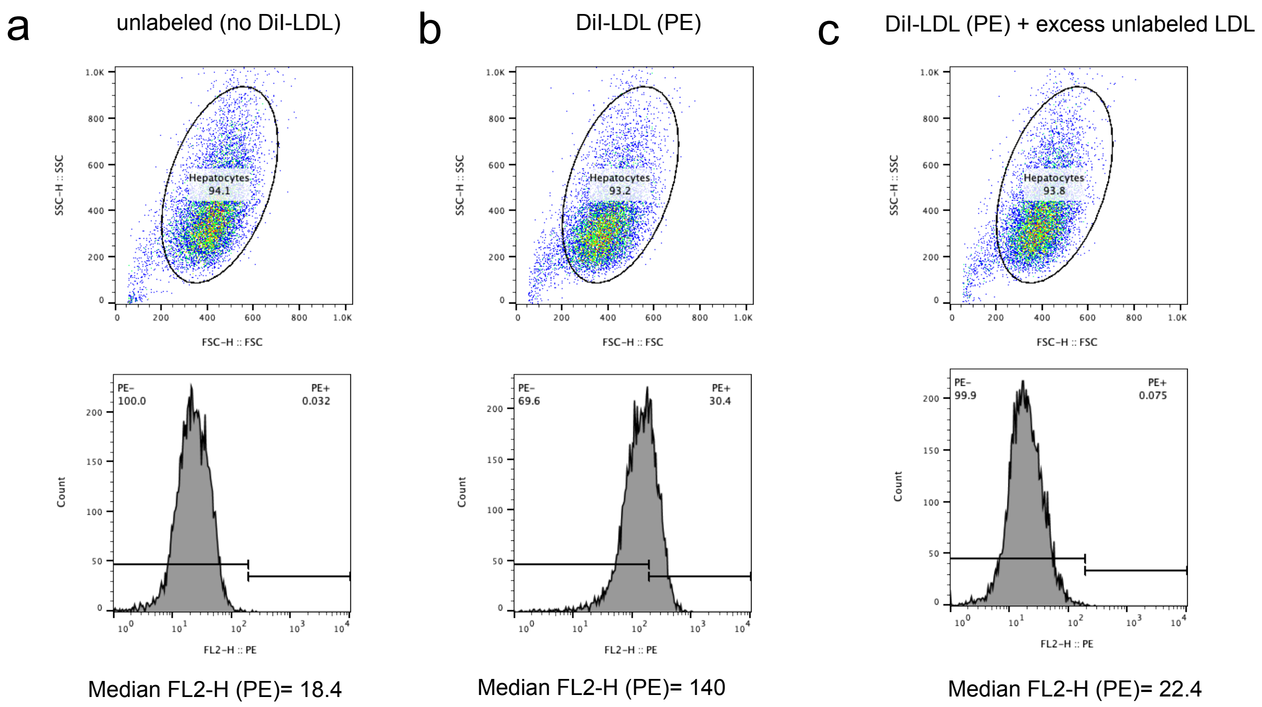

Supplementary Fig. 9: Representative gating strategy used for DiI-LDL uptake and binding experiments. (a–c) For DiI-LDL uptake and binding experiments, cells were washed and incubated in fresh media containing DiI-LDL (30 μg cholesterol/ml) for 2-8 h at 37 °C (uptake) or 4 °C (binding). Non-specific uptake was determined in extra wells containing a 50-fold excess of unlabeled native LDL. At the end of the incubation period, cells were washed twice and resuspended in 1 ml of PBS for FACS. Samples were collected on a BD FACSCalibur and analyzed using WINMDI v2.8 or FlowJo v10. Live cells were enriched using FSC/SSC. 10,000 events were collected for each sample. Cells incubated without DiI-LDL (FL2-H, PE) were used to set positivity for PE. Results are expressed in terms of specific median intensity of fluorescence (MFI) after subtracting out autofluorescence of cells incubated in the absence of DiI-LDL and correcting for non-specific uptake/binding by subtracting out the fluorescence of cells incubated with DiI-LDL and an excess of unlabeled LDL.

Supplementary Table 1: Primer Sequences

Cloning Primers		
**Name**	**Forward (5'-3')**	**Reverse (5'-3')**
**MMAB/MVK Promoter**	AAGAGCTCAGACACCAGTAGGACT TCTGACAACATGG	AAGAGCTCGAGCCAGGCTGCTTGACGG
**Mouse qPCR Primers**		
**Gene Name**	**Forward (5'-3')**	**Reverse (5'-3')**
18S	AGCTATCAATCTGTCAATCCTGTC	GCTTAATTTGACTCAACACGGGA
LDLR	AGTTGGCTGCGTTAATGTGAC	TGATGGGTTCATCTGACCAGT
HMGCR	GTCATTCCAGCCAAGGTTGT	GGGACCACTTGCTTCCATTA
SREBP2	GATGCACAAGTCTGGCGTTC	CATTGTCCACCAGACTGCCT
CYP51A	GAAACGCAGACAGTCTCAAGA	ACGCCCATCCTTGTATGTAGC
PCSK9	GGAGCTGGCCTTGAAGTTGCC	ACCGTGGAGGGGTAATCCGC
MMAB	CATTGCAGGACGTCGGCTCG	AGGCACCACACGTCTCTCGG
MUT	TGGGTTTGCCAACTGTGAAAA	GGTATTCCCTCAGCTACAGCTT
DHCR24	GCAGGTGCGGGAATGGAAGG	TGGAGGTCAGCAGGGCAGTC
MVK	CATGGCAAGGTAGCACTGG	GATACCAATGTTGGGTAAGCTGA
VPS41	AGTGGCCAAGGAACGAGACCA	TCTGGCATTTGCGTGCTGCT
SLC6A4	TGCTTCGATCAGCGCGACAG	TGGACAGCACGTTTGCAGGC
YIF1A	ACAGGAGCAGACGTGGCCTT	AACGGTGCAGCTCCTTGTGC
UBOX5	AGCACAGACGGCATTGGCAG	GTGGGACAGTGGACCTGTCGAG
TMEM14A	GGACTCCAGGTACCGCGCTT	CGG AACACCACCTCTCCGCT
**Mouse qPCR Primers**		
**Gene Name**	**Forward (5'-3')**	**Reverse (5'-3')**
18s	TTCCGATAACGAACGAGACTCT	TGGCTGAACGCCACTTGTC
Mmab	CTCAGCTATTGGGTTTGCCAT	GTGTGCTTTAAGTGAGCCTCC
Mvk	CGGCCTTGAACTTGAGAACT	CCTTGCTCAAGAAAGCTCGT
