# Supplementary material for: MMAB promotes negative feedback control of cholesterol homeostasis
Source: Nat Commun. 2021 Nov 8;12:6448. doi: 10.1038/s41467-021-26787-7 (PMC8575900; doi:10.1038/s41467-021-26787-7)
Supplement: Supplementary file 3 — Description of Additional Supplementary Files [file 41467_2021_26787_MOESM3_ESM.docx]

**DESCRIPTION OF ADDITIONAL SUPPLEMENTARY FILES**

# MMAB promotes negative feedback control of cholesterol homeostasis

Leigh Goedeke^1,2^, Alberto Canfrán-Duque^1,3^, Noemi Rotllan^1,3^, Balkrishna Chaube^1,3^, Bonne M. Thompson^4^, Richard G. Lee^5^, Gary W. Cline^2^, Jeffrey G. McDonald^4^, Gerald I. Shulman^2,6^, Miguel A. Lasunción^7^, Yajaira Suárez^1,3^, and Carlos Fernández-Hernando^1,3,#^

^1^Vascular Biology and Therapeutics Program, Yale School of Medicine

^2^Department of Internal Medicine, Yale School of Medicine,

^3^Integrative Cell Signaling and Neurobiology of Metabolism Program, Department of Comparative Medicine and Pathology, Yale School of Medicine, New Haven CT 06520, USA

^4^Center for Human Nutrition. University of Texas Southwestern Medical Center, Dallas TX 75390, USA.

^5^Cardiovascular Group, Antisense Drug Discovery, Ionis Pharmaceuticals, Carlsbad, CA, 92010, USA.

^6^Department of Cellular & Molecular Physiology, Yale School of Medicine

^7^Servicio de Bioquímica-Investigación, Hospital Universitario Ramón y Cajal, Instituto Ramón y Cajal de Investigación Sanitaria (IRyCIS) and CIBER de Fisiopatología de la Obesidad y Nutrición (CIBERobn), Madrid Spain.

^#^**Correspondence:** carlos.fernandez@yale.edu

**This PDF file contains:**

Supplementary Dataset Descriptions

**File Name:** Supplementary Data 1

**Description:** Top candidate genes from the genome-wide RNAi screen whose knockdown either increased or decreased LDLR activity (robust z-score of £ –2.0 or ³ 2.0) are listed in tab ‘siRNA Screen Hits.’ Top candidate genes whose expression was also significantly modulated by intracellular cholesterol levels and/or had SNPs previously linked to alterations in plasma cholesterol/CVD risk can be found under tab ‘Top 250 Hits.’ Functional annotation clustering of the top 250 genes was performed using DAVID and used to identify functional interactions between candidate genes using STRING. Raw clustering data for this analysis can be found under tabs ‘DAVID Functional Clustering 250’ and ‘STRING 250 Hits,’ with main clustering nodes outlined in subsequent tabs. This Dataset is related to **Fig. 1–2** and **Supplementary Fig. 1.**

**File Name**: Supplementary Data 2

**Description:** Gene expression analysis of Huh7 cells cultured in DMEM containing 10% FBS and supplemented with 120 µg/ml native LDL (nLDL, to cholesterol enrich) or 5 µM statin (to cholesterol deplete) or vehicle control for 24 h. Those genes significantly modulated by statin and/or nLDL treatment are listed in subsequent tabs (*P* £ 0.05*,* FC ³1.1 or £ –1.1). Raw data for the functional annotation clustering of up- and down-regulated genes using DAVID can also be found here. This Dataset is related to **Fig. 1.**

**File Name:** Supplementary Data 3

**Description:** Top candidate genes listed in the tab ‘Deconvolute Screen Hits’ were subjected to further validation by undergoing a second microscope-based screening assay with 3 individual siRNAs from the initial siRNA pool used in the primary RNAi screen. Raw data for the heatmap in **Supplementary Fig. 2** can be found under tab ‘Deconvolute Screen Heatmap. This Dataset is related to **Supplementary Fig. 2.**

**File name:** Supplementary Data 4

**Description:** Untargeted global metabolic analysis of Huh7 cells transfected with an siRNA against MMAB (MMAB_1, 4, 5) or a non-silencing control siRNA (NS_1-5). Raw metabolite data normalized to total protein is listed under tab ‘ProNormImpData(Huh7). This Dataset is related to **Fig. 5.**

**File name:** Supplementary Data 5

**Description:** Untargeted global metabolic analysis of wild-type (WT_1-5) and *MUT* (Mut_1-5) fibroblasts incubated in DMEM containing 10% LPDS for 24 h. Raw metabolite data normalized to total protein is listed under tab ‘ProNormImpData(fb). This Dataset is related to **Fig. 5.**

**File name:** Supplementary Data 6

**Description:** Hepatic gene expression analysis of mice treated with 50 mg/kg Control ASO or MMAB ASO. Transcripts significantly modulated by MMAB knockdown (*P* £ 0.05*,* FC ³1.5 or £ – 1.5) are listed under tab ‘Fold-change’ (MMAB ASO vs. CON ASO). The top modulated pathways can be found in subsequent tabs. This Dataset is related to **Fig. 7.**
