# Supplementary material for: MMAB promotes negative feedback control of cholesterol homeostasis
Source: Nat Commun. 2021 Nov 8;12:6448. doi: 10.1038/s41467-021-26787-7 (PMC8575900; doi:10.1038/s41467-021-26787-7)
Supplement: Supplementary file 10 — Reporting Summary [file 41467_2021_26787_MOESM10_ESM.pdf]

nature research

Corresponding author(s): Carlos Fernandez-Hernando

Last updated by author(s): Sep 13, 2021

Reporting Summary

Nature Research wishes to improve the reproducibility of the work that we publish. This form provides structure for consistency and transparency in reporting. For further information on Nature Research policies, see Authors & Referees and the Editorial Policy Checklist .

Statistics

For all statistical analyses, confirm that the following items are present in the figure legend, table legend, main text, or Methods section.

n/a	Confirmed	
☐	☒	The exact sample size (*n*) for each experimental group/condition, given as a discrete number and unit of measurement
☐	☒	A statement on whether measurements were taken from distinct samples or whether the same sample was measured repeatedly
☐	☒	The statistical test(s) used AND whether they are one- or two-sided<br>*Only common tests should be described solely by name; describe more complex techniques in the Methods section.*
☐	☒	A description of all covariates tested
☐	☒	A description of any assumptions or corrections, such as tests of normality and adjustment for multiple comparisons
☐	☒	A full description of the statistical parameters including central tendency (e.g. means) or other basic estimates (e.g. regression coefficient) AND variation (e.g. standard deviation) or associated estimates of uncertainty (e.g. confidence intervals)
☐	☒	For null hypothesis testing, the test statistic (e.g. *F*, *t*, *r*) with confidence intervals, effect sizes, degrees of freedom and *P* value noted<br>*Give P values as exact values whenever suitable.*
☒	☐	For Bayesian analysis, information on the choice of priors and Markov chain Monte Carlo settings
☒	☐	For hierarchical and complex designs, identification of the appropriate level for tests and full reporting of outcomes
☐	☒	Estimates of effect sizes (e.g. Cohen's *d*, Pearson's *r*), indicating how they were calculated

Our web collection on statistics for biologists contains articles on many of the points above.

Software and code

Policy information about availability of computer code

Data collection	Western blot acquisition: Odyssey Infrared Imaging System (LI-COR Biotechnology)<br>DiI-LDL uptake and binding experiments: BD FACSCalibur, EVOS digital inverted florescence microscope (AMG), Leica SP5 II confocal microscope equipped with a 63X Plan Apo Lense<br>RNAi screen transfection and image acquisition: Wellmate Microplate Dispenser (Matrix Technologies), BioTek Plate Washer (PerkinElmer), Arrayscan VTI HCS Reader (Thermo Scientific) with a Zeiss 10x objective, BioApplication's Target Activation v3 (Thermo Scientific)<br>qPCR: iCycler Real-Time Detection System (Biorad)<br>Mass spec: AB Sciex QTrap 6500, Agilent GC (HP6890)-MS (HP5973)
Data analysis	Statistical analysis: GraphPad Prism Software Version 7.0a<br>FACS analysis: WINMDI v2.8 and FlowJo v10<br>Affymetrix array data analysis: GeneSpring GX software version 11.5 (Agilent Technologies)<br>RNAi screen bioinformatic analysis: DAVID, STRING v9.05, Cytoscape plugin v3.4, EnrichmentMap plugin (v3.0), UCSC Genome Browser (NCBI36/hg18, http://genome.ucsc.edu)<br>Fluorescent/Western blot image analysis/quantification: ImageJ (NIH), Adobe Photoshop CS5<br>RNAseq analysis: PartekFlow® software, version 8.0.19.0405 (Partek, Inc., St. Louis, MO), STAR algorithm (mmu10), Ingenuity Pathway Analysis Spring Release 2019 (Ingenuity Systems QIAGEN, Redwood City, CA, USA), Qlucore Omics Explorer v 3.2 (Qlucore AB, Lund, Sweden)

For manuscripts utilizing custom algorithms or software that are central to the research but not yet described in published literature, software must be made available to editors/reviewers. We strongly encourage code deposition in a community repository (e.g. GitHub). See the Nature Research guidelines for submitting code & software for further information.

Data

Policy information about availability of data

All manuscripts must include a data availability statement. This statement should provide the following information, where applicable:

- Accession codes, unique identifiers, or web links for publicly available datasets
- A list of figures that have associated raw data
- A description of any restrictions on data availability

RNA-seq data and Affymetrix array data that support the findings of this study have been deposited in the Gene Expression Omnibus under accession code GSE169712 (https://www.ncbi.nlm.nih.gov/geo/query/acc.cgi?acc=GSE169712) and GSE183790 (https://www.ncbi.nlm.nih.gov/geo/query/acc.cgi?acc=GSE183790), respectively. RNAi screen data that support the findings of this study have been deposited in PubChem with the Assay ID 1671195 (https://pubchem.ncbi.nlm.nih.gov/bioassay/1671195). All other data that support the findings of this study are within the article and its Supplementary Information and Source Data files.

Field-specific reporting

Please select the one below that is the best fit for your research. If you are not sure, read the appropriate sections before making your selection.

[x] Life sciences [ ] Behavioural & social sciences [ ] Ecological, evolutionary & environmental sciences

For a reference copy of the document with all sections, see nature.com/documents/nr-reporting-summary-flat.pdf

Life sciences study design

All studies must disclose on these points even when the disclosure is negative.

Sample size	Animal sample size for each study was chosen based on literature documentation of similar well-characterized experiments (Rayner et al., 2010; Shimomura et al., 1999; Horton et al., 1998; Mattison et al., 2014; Goedeke et al 2015). Sample sizes for cell culture experiments (at least three biological replicates) were chosen based on extensive in vitro experience in the lab. Sample sizes are listed in the figure legends and main text.
Data exclusions	For siRNA experiments, knockdown was assessed prior to downstream analyses (gene expression, LDL uptake/binding, metabolomics etc). Any biological replicate that did not achieve ≥80% knockdown was excluded from subsequent analyses. For all other in vitro experiments, no data was excluded. No animals were excluded from this study.
Replication	In vitro experiments were routinely repeated independently at least in three biological replicates. Key cell culture experiments (knockdown of MMAB, dil-LDL uptake/binding, and assessment of SREBP2 gene expression) were replicated successfully by at least two investigators in the lab. Multiple mice (n = 3-8 per group) were used in all in vivo experiments. All attempts at replication were successful.
Randomization	Mice were weight-matched and randomized to treatment groups before the start of injections by an independent investigator who did not participate in subsequent analyses. For in vitro experiments, cells were randomly assigned to groups to avoid bias.
Blinding	When possible, blinding was performed during data collection and analysis for in vitro and in vivo experiments. For experiments in which investigators could not be blinded for practical reasons (i.e during treatment of mice and cells), results were independently analyzed by another investigator.

Reporting for specific materials, systems and methods

We require information from authors about some types of materials, experimental systems and methods used in many studies. Here, indicate whether each material, system or method listed is relevant to your study. If you are not sure if a list item applies to your research, read the appropriate section before selecting a response.

Materials & experimental systems

n/a	Involved in the study	
[ ]	[x]	Antibodies
[ ]	[x]	Eukaryotic cell lines
[x]	[ ]	Palaeontology
[ ]	[x]	Animals and other organisms
[x]	[ ]	Human research participants
[x]	[ ]	Clinical data

Methods

n/a	Involved in the study	
[x]	[ ]	ChIP-seq
[ ]	[x]	Flow cytometry
[x]	[ ]	MRI-based neuroimaging

Antibodies

Antibodies used	The following antibodies were used in this study: LDLR (Abcam #ab30532, 1:1000), MMAB (Novus #NBP1-86602, 1:1000), HSP90 (BD Biosciences #610418, 1:1,000), CYP51A (Proteintech #13431-1-AP, 1:500), ACTIN (Abcam #ab8227, 1:1000), DHCR24 (Cell Signaling #2033, 1:1000), PCSK9 (Cayman Chemical #10240, 1:1000), SREBP2 (BD Biosciences #557037, 1:500), HMGCR (culture supernatant of the mouse hybridoma, A9 cell line [ATCC #CRL-1811], 1:10), LDLR (Cayman Chemical #10007665; 1:1000), NPC1 (Novus #NB400-148, 1:500), fluorescently labeled secondary antibodies (Invitrogen #A10043, #A21057; 1:5000) and HRP-conjugated secondary antibodies (Cell Signaling Technologies #7074, #7076; 1: 5000).
Validation	All Cell Signaling, Cayman Chemical, Abcam, Novus, BD Bioscience, Proteintech and Invitrogen antibodies were validated by the manufacturer for Western blotting use; validation can be found on the manufacturers' website. The mouse hybridoma, A9 cell line has previously been validated by Millipore Sigma (https://www.emdmillipore.com/US/en/product/Anti-HMG-CoA-Reductase-Antibody-clone-IgG-A9,MM_NF-MABS1233).<br>Antibodies were also independently validated in the laboratory using the following experiments:<br>1) The MMAB antibody was validated in Huh7 cells, Hepa cells and mouse liver tissue using an siRNA against MMAB or animals injected with antisense oligonucleotides against MMAB or non-silencing control.<br>2) LDLR and NPC1 antibodies were validated in Huh7 cells transfected with an siRNA against LDLR, NPC1 or non-silencing control siRNA.<br>3) Antibodies against the SREBP2-responsive genes (LDLR, DHCR24, CYP51A1, HMGCR, SREBP2, PCSK9) were validated in hepatic cells incubated in LPDS, LPDS + statin, and/or LPDS + LDL, conditions which are known to increase and decrease the expression of these genes, respectively.

Eukaryotic cell lines

Policy information about cell lines

Cell line source(s)	Age and sex-matched fibroblasts from patients with methylmalonic aciduria due to MUT deficiency (MUT fibroblasts, #GM00050) and otherwise healthy individuals (WT fibroblasts, #GM05659) were obtained from the NIGMS Human Genetic Cell Repository (Coriell Institute for Medical Research). All cells obtained from the NIGMS Human Genetic Repository were de-identified and are not considered to be human subjects. The human hepatocellular carcinoma cell line, Huh7 (JCRB Cell Bank JCRB0403), and mouse hepatic cell line (Hepa1¬–6; ATCC CRL-1830) were a kind gift from Dr. Edward Fisher (NYU School of Medicine). HeLa cells were purchased from ATCC (ATCC CCL-2).
Authentication	All primary fibroblast cells were authenticated by Coriell Institute for Medical Research and used within 2-3 passages. Stable cell lines (Huh7, Hepa1-6, HeLa) were authenticated by morphology check at high and low plating densities, growth curve analysis, and periodic assessment of mycoplasma infection.
Mycoplasma contamination	All cell lines tested negative for mycoplasma contamination.
Commonly misidentified lines<br>(See ICLAC register)	No commonly misidentified cell lines were used in this study.

Animals and other organisms

Policy information about studies involving animals; ARRIVE guidelines recommended for reporting animal research

Laboratory animals	C57BL/6J (Wild-type, WT): Jackson Laboratories (#000664), male, age 8 weeks<br>Apobec1-/-;Ldlr-/+): kind gift from Dr. Daniel Rader (UPenn), male, age 8 weeks
Wild animals	No wild animals were used in this study.
Field-collected samples	No field-collected samples were used in this study.
Ethics oversight	All animal maintenance and experiments were performed in accordance with NIH guidelines and were approved by the Institutional Animal Care Use Committee of Yale University School of Medicine.

Note that full information on the approval of the study protocol must also be provided in the manuscript.

Flow Cytometry

Plots

Confirm that:

- [x] The axis labels state the marker and fluorochrome used (e.g. CD4-FITC).
- [x] The axis scales are clearly visible. Include numbers along axes only for bottom left plot of group (a 'group' is an analysis of identical markers).
- [x] All plots are contour plots with outliers or pseudocolor plots.
- [x] A numerical value for number of cells or percentage (with statistics) is provided.

Methodology

Sample preparation	Huh7 or Hepa cells were transfected in 6-well plates with an siRNA against MMAB or non-silencing control siRNA for 48 h. For DiI-LDL uptake and binding experiments, cells were washed and incubated in fresh media containing DiI-LDL (30 ug cholesterol/ml) for 2-8 h at 37C (uptake) or 4C (binding). Non-specific uptake was determined in extra wells containing a 50-fold excess of unlabeled native LDL. At the end of the incubation period, cells were washed twice and resuspended in 1 ml of PBS for Flow Cytometry analysis.
Instrument	BD FACSCalibur
Software	WINMDI v2.8 and FlowJo v10
Cell population abundance	Live cells were enriched using FSC/SSC. 10,000 events were collected for each sample.
Gating strategy	Live cells were gated using FSC and SSC. Cells incubated without diI-LDL (PE) were used to set positivity for DiI-LDL (PE). The results are expressed in terms of specific median intensity of fluorescence (MFI) after subtracting out autofluorescence of cells incubated in the absence of DiI-LDL and correcting for non-specific uptake by subtracting out the fluorescence of cells incubated with DiI-LDL and and an excess of unlabeled LDL.

- [x] Tick this box to confirm that a figure exemplifying the gating strategy is provided in the Supplementary Information.
